# Investigation and Validation of Molecular Characteristics of Endometrium in Recurrent Miscarriage and Unexplained Infertility from a Transcriptomic Perspective

**DOI:** 10.7150/ijms.69648

**Published:** 2022-03-06

**Authors:** Yuxin Ran, Jie He, Ruixin Chen, Yan Qin, Zheng Liu, Yunqian Zhou, Nanlin Yin, Hongbo Qi, Wei Zhou

**Affiliations:** 1 Department of Obstetrics, The First Affiliated Hospital of Chongqing Medical University, Chongqing 400016, China.; 2 Chongqing Key Laboratory of Maternal and Fetal Medicine, Chongqing Medical University, Chongqing 400016, China.; 3 Joint International Research Laboratory of Reproduction and Development of Chinese Ministry of Education, Chongqing Medical University, Chongqing 400016, China.; 4 Department of Gynecology and Obstetrics, West China Second Hospital, Sichuan University, Chengdu 610041, China.; 5 Department of Gynecology, the First Affiliated Hospital of Chongqing Medical University, Chongqing 400016, China.; 6 Center for Reproductive Medicine, The First Affiliated Hospital of Chongqing Medical University, Chongqing 400016, China.; 7 Department of Obstetrics, Chongqing Health Center for Women and Children, Chongqing 401147, China.

**Keywords:** Recurrent miscarriage, Unexplained infertility, Molecular characteristics, Endometrium, Transcriptome analysis.

## Abstract

Recurrent miscarriage (RM) and unexplained infertility (UI) are gordian knots in reproductive medicine, which are troubling many patients, doctors, and researchers. Although these two diseases of early pregnancy have a significant impact on human reproductive health, little is known about the specific mechanisms, which caused treatment difficulties. This study focused on the molecular signatures underlying the pathological phenotypes of two diseases, with the hope of using statistical methods to identify the significant core genes. An unbiased Weighted Correlation Network Analysis (WGCNA) algorithm was used for endometrial transcriptome data analysis and the disease-related gene modules were screened out. Through enrichment analysis of the candidate genes, we found similarities between both diseases and shared enrichment of immune-related pathways. Therefore, we used immune algorithms to assess the infiltration of immune cells and found abnormal increases of CD8+T cells and neutrophils. In order to explore the molecular profile behind the immunophenotypic changes, we used the SVM algorithm and LASSO regression to identify the core genes with diagnostic capacity in both diseases and discussed their significance of immune disorders in the endometrium. In the end, the satisfactory diagnostic ability of these core genes was verified in the broader group. Our results demonstrated the presence of immune disorders in non-pregnancy tissues of RM and UI, and identified the core molecules of this phenotype, and discuss mechanisms. This provides exploratory evidence for the in-depth understanding of the mechanism of RM and UI and may provide potential targets for their future treatment.

## 1. Introduction

Pregnancy is a delicate and complex process, from the beginning of fertilization to the end of childbirth, and our understanding of pregnancy is still very preliminary. The majority of pregnancy failure occurs in the early stages of pregnancy and manifests as infertility or miscarriage depending on the stage of embryo termination 1. Infertility, a common disease with an estimated incidence of 8-12% worldwide, refers to the diagnosis of attempted conception without success for more than 12 months 2, 3. According to the WHO report, one in four couples in developing countries is found to be infertile, posing a heavy social burden 4, 5. Unfortunately, nearly a third of infertility patients are unaware of the cause, known as unexplained infertility (UI) 2. Compared to UI, miscarriage seems to be associated with more serious complications, such as hemorrhage and infection. Recurrent miscarriage (RM) refers to the occurrence of two or more consecutive spontaneous miscarriage, with an approximate incidence of 2.6% worldwide 1, 6. Importantly, the psychological, social, and physical burden on women who suffers from RM is significant, for example, up to 41% of women experience clinical anxiety or even post-traumatic stress disorder after a pregnancy loss. 7. In brief, both diseases which occur in the early stage of pregnancy, cause physical and mental suffering to many women and impose a heavy health and economic burden on society 1.

Due to the lack of understanding of the pathogenesis, the treatment of UI and RM has been a thorny challenge in the field of reproductive medicine. While according to the current research, as the most critical factor for maternal acceptance of the embryo, the role of endometrium in pregnancy failure has been gradually recognized by researchers 8, 9. Under the stimulation of progesterone, endometrial stromal cells expand morphologically in an exuberant-metabolism appearance with the accumulation of glycogen and lipid droplets in the expanding cytoplasm 10. Meanwhile, the number of NK cells in the endometrium also shows periodic changes, reaching the proportion of 30%-40% of the cells in the endometrium matrix after ovulation, playing a crucial immunosuppressive function and providing a receptive immune environment for the embryo 9. There is now a consensus that endometrial lesion, especially the lack of an immunosuppressive environment has been shown to be one important reason for adverse pregnancy outcomes such as infertility and miscarriage 8, 11. Research indicated that the disruption of natural killer (NK) cells in the endometrium is highly correlated to the occurrence of infertility and pregnancy 12, 13. Treg cells regulate the immune response to fetal antigens and enhance uterine receptivity and evidence indicated that the disorder in number and function of Treg cells are also important mechanisms of pregnancy failure 14. Immunosuppressive drugs are used in clinical immunotherapy, for example, progesterone and prednisolone were used to improve the endometrium receptivity and promote the maintenance of pregnancy 15, 16. However, the latest review reports that these treatments are only effective for a part of patients, and do not show satisfactory results at the overall level 17. This suggests that we need to further understand the heterogeneity of decidual tissue in susceptible women, especially in view of the molecular expression imbalance, and to classify these patients based on their difference for optimal treatment outcomes.

High-throughput sequencing technology is a potent tool for exploring molecular expression patterns and has been applied to the study of various diseases since its inception. Due to its advantage of understanding the connections between genes from a global perspective, it provides a powerful help to explain the molecular characteristics of pathological tissues. For example, for preeclampsia, a complex disease of pregnancy, transcriptome analysis has provided detailed expression information of all genes in the affected women and further screened the network of the risk genes most relevant to symptom onset, thus revealing the core biological alterations of this disease and providing precise targets for therapeutic strategies 18, 19. Similarly, the high throughput method was also used for exploring the pathogenesis of UI and RM, especially using genome sequencing and exon sequencing methods, aiming to find genetic variations associated with disease 20-23. A recent single-cell study showed an increase in CD8+T cells and activation of NK cells in the decidua of RM patients and depicted the molecular atlas 24. However, this research did not reveal the characteristics of the endometrium in these women during non-pregnancy, and the disease may occur due to intrinsic changes in the endometrium, and the effects of pregnancy hormones may mask such gene expression abnormalities. Given this transition of the endometrium during pregnancy, any single analysis of endometrium or decidua is partial, suggesting the need for a complete view of this complex tissue. Transcriptome studies on the endometrium of RM and UI have provided massive information on biological molecules, however, effective algorithms are still needed to explore the intricate molecular networks and find the core basis of RM and UI 25-27.

As sequencing has evolved, so has the soft power of statistical analysis, broadening our understanding of vast amounts of biological data. The Weighted Correlation Network Analysis (WGCNA) is an algorithm suitable for multiple samples, which clusters genes with similar expression patterns into modules and analyzes the relationship between these gene clusters and clinical information 28. At present, this algorithm has been widely used in the analysis of transcriptome data and has provided great help for the screening of biomarkers 29, 30. Different from the traditional analysis methods that focus on the association between individual genes and disease characteristics, WGCNA focuses more on the association between genes, which makes up for the shortcomings of transcriptome data analysis and improves the understanding of disease occurrence. Especially for endometrium, a tissue with complex cellular composition, and diseases with multiple etiologies of RM and UI, WGCNA is more suitable for finding genes with key roles from massive data.

Based on our understanding of human reproductive failure, we hypothesize that disorder of endometrial function in women is a common cause of RM and UI. To further understand the abnormality based on gene expression in female endometrium with RM and UI, sequencing results of endometrium from public databases were analyzed. In combination with the current research progress and the statistical analysis of sequencing data, we depicted the molecular atlas of the endometrium of RM and UI, and analyzed the similarities and differences between the two diseases, aiming to provide a new understanding of reproductive failure in the early stages of pregnancy.

## 2. Methods

### 2.1 Data collection

Endometrial tissue RNA expression data were searched in and downloaded from the NCBI Gene Expression Omnibus database (GEO) (http://www.ncbi.nlm.nih.gov/geo/) and the Sequence Read Archive (SRA) database (https://www.ncbi.nlm.nih.gov/sra). The following inclusion criteria were utilized: (1) sample donors include normal controls, RM, and UI women of reproductive age. The judgment criteria of RM and UI follow the 2018 ESHRE guidelines and 2019 CFAS guidelines, respectively^31^, ^32^; (2) endometrium at mid-secretion (i.e., the window period of embryo implantation); (3) without uterine malformations, fallopian tube blockage, obvious endocrine disorders, karyotype abnormalities, acute infections, and other comorbidities; (4) data generated by RNA-Seq or Microarray techniques.

After screening, the datasets GSE165004 (24 NC, 24 RM, and 24 UI), GSE26787 (5 NC and 5 RM), GSE65099 (10 RM), SRP071561 (3 NC and 6 RM), GSE144895 (20 UI), GSE58144 (72 NC and 43 UI), and GSE4888 (20 NC) were included in this study. Among them, data of 12 NC, 12 RM, and 12 UI from GSE165004 were used for analyzing and screening, total data (72 samples) of GSE165004 were used for internal validation, and GSE26787, GSE65099, SRP071561, GSE144895, GSE58144, and GSE4888 were used for external validation.

The microarray data were were quantile normalized and the RNA-seq data were normalized by transcripts per million (TPM) method. The batch effects between different datasets were removed by ranking and weighting algorithms.

### 2.2 Weighted Correlation Network Analysis (WGCNA)

Using the normalized mRNA expression data (calculated by the R package “limma”) as input files, WGCNA was completed using the R package "WGCNA" to reveal the coexpression of genes and the strength of the relationship between gene modules and clinical phenotypes (NC, RM, and UI) 28. This analysis mainly consists of the following steps: (1) The gene co-expression network was constructed by calculating the correlation coefficients between genes. (2) The proper soft-thresholding power was selected using the “pickSoftThreshold” function, to generate the adjacency matrix. (3) This matrix was then used to create a topological overlap matrix (TOM) to determine the similarity of expression between genes. (4) Hierarchical clustering tree (average linkage hierarchical clustering) was constructed based on weighted correlation coefficients between genes, and then different gene modules were obtained using the dynamic tree cut algorithm (minModuleSize = 20). The similar modules were merged when mergeCutHeight = 0.25. (5) The correlation between gene modules and clinical phenotypes (NC, RM, and UI) was assessed using the Pearson correlation coefficient.

### 2.3 Functional enrichment analysis

Reactome (http://reactome.org) is a database of biological pathways that contains a large amount of reliable and detailed annotation information on biological processes, reactions, and pathways in humans 33. Based on this database, we performed the enrichment analysis using the R package “clusterProfiler” to identify and annotate the functional activities involved in the genes associated with different clinical phenotypes 34. The entries with a P-value > 0.05 were considered significant and then visualized using the R package “ggplot2” 35.

### 2.4 Gene set enrichment analysis (GSEA)

The GSEA software (version 4.0.3) provided by the Broad Institute (http://www.broadinstitute.org/gsea) was used to perform this analysis and all of the steps were completed using default parameters. The gene sets of inflammatory response were downloaded from the Molecular Signatures Database (http://software.broadinstitute.org/gsea/msigdb). Finally, the inflammatory response was considered activated when the normalized enrichment score (NES) generated was greater than zero.

### 2.5 Immune cell abundance estimation

The relative levels of immune cells in endometrial tissue were calculated using the Immune Cell Abundance Identifier (ImmuCellAI) 36. This is an efficient tool for extracting information on the abundance of 24 immune cells from RNA-Seq count or microarray data based on the ssGSEA algorithm. This analysis was completed with the default parameters and the results were visualized using the R package "ggplot2".

### 2.6 Variable filtering

The Support vector machines (SVM) and LASSO regression analysis were used to screen the core genes of RM and UI. Firstly, the supervised classification algorithm SVM was performed to estimate the value of genes to classification using the R package “e1071” and “caret” 37, 38. The genes with P-value > 0.05 were selected and ranked by importance. Secondly, the core genes were identified from these genes using the LASSO algorithm 39. In this step, the regression model with 10-fold cross-validation was constructed using the R package “glmnet”. The parameter “family” was set as“binomial”, and the best lambda value was selected by “lambda.1se”.

### 2.7 Statistical analysis

Statistical analyses were conducted using the SPSS (version 25.0, Chicago, IL, USA) software. The comparisons of multiple groups were performed by one-way ANOVA and then an LSD-t test. The threshold for statistical significance was set at p<0.05. The R package "ggplot2" was employed to visualize the results.

## 3. Results

### 3.1 The key gene modules involved in RM and UI

**Figure 1:**
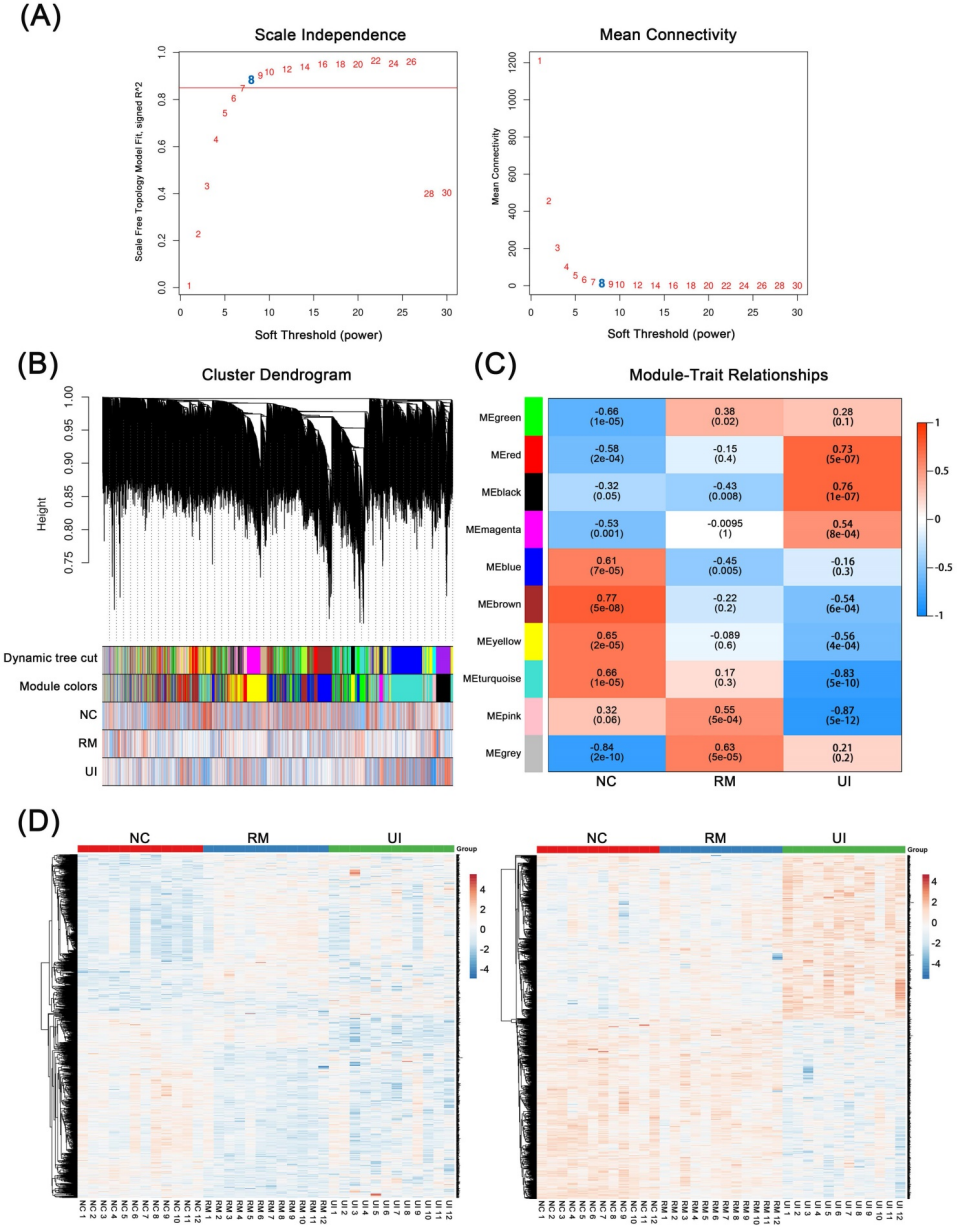
** Weighted Co-Expression Network Construction and Identification of Key Modules.** (A) Determination of soft-threshold power in the WGCNA. (B) Clustering dendrograms showing 10 modules containing highly connected genes. (C) Heatmap of the correlation between the modules and clinical diagnosis. (D) Heatmap of the gene expression in the candidate modules of RM group (left panel) and UI group (right panel). (NC: n = 12; RM: n = 12; UI: n = 12)

WGCNA was performed based on mid-secretory endometrial mRNA expression profiles to reveal genes underlying RM and UI, respectively. By calculating the scale-independence and the mean connectivity, the power value of 8 (R2 = 0.85) was screened as the optimal soft threshold to establish a scale-free network (Fig. 1A). On this basis, these genes are grouped into different sets according to the correlation of expression. Moreover, these sets were further clustered and a total of 10 gene modules were obtained (Fig. 1B). Subsequently, module-trait relationship analysis revealed the association between these gene modules and clinical phenotypes. As shown in Fig. 1C, the pink (P < 0.001) and gray (P < 0.001) modules are significantly positively correlated with RM, while the blue module (P = 0.005) are significantly negatively correlated. Meanwhile, the red and dark modules were highly positively correlated with UI, and the turquoise and pink modules were highly negatively correlated (P < 0.001 for each of them). In addition, the brown and yellow modules were strongly associated with the normal endometrial phenotype (P < 0.001 for each of them). Taken as a whole, the genes contained in the candidate modules of RM (n = 1325) and UI (n = 1434) have noticeably different expression patterns in different clinical phenotypes (Fig. 1D). However, 29 of the upregulated genes in RM were also upregulated in the UI, while 8 of the downregulated genes in RM were also downregulated in the UI (the data not shown). This suggests that there are still some potential connections between the two different clinical phenotypes, RM and UI.

### 3.2 Multiple biological processes were changed in RM and UI

**Figure 2:**
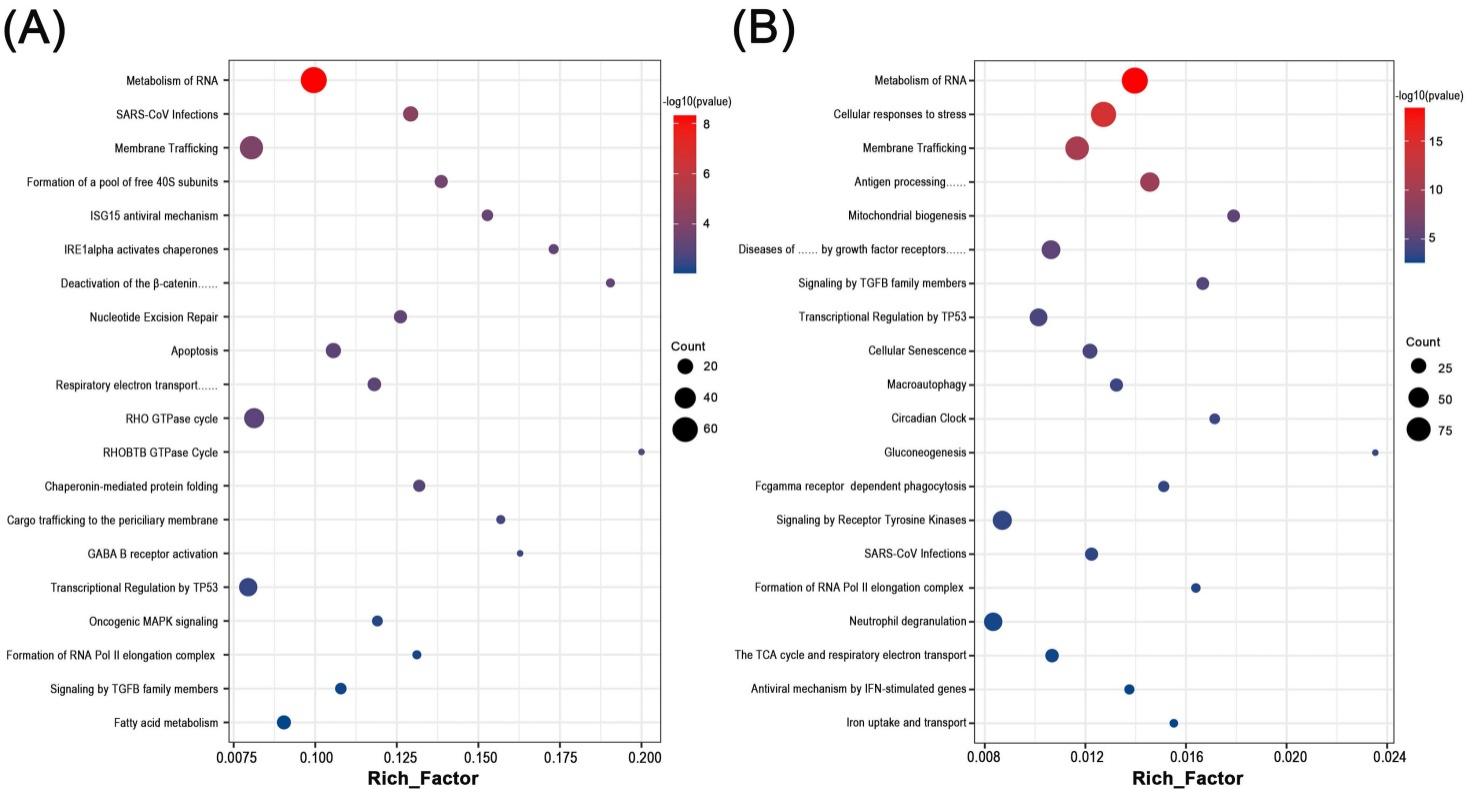
** Biological function enrichment analysis of genes in candidate modules.** Dot plot of biological function enrichment of the genes in RM candidate modules (A) and UI candidate modules (B).

By functional annotation and enrichment analysis of these candidate genes, we sought to explore the potential biological changes in the endometrium of RM and UI. Interestingly, six of the top 20 processes involved in each of RM and UI were identical: Metabolism of RNA, SARS-CoV infections, membrane trafficking, transcriptional regulation by TP53, formation of RNA Pol II elongation complex, and signaling by TGFB family members. Besides, the RM-related genes were enriched in the ISG15 antiviral mechanism, apoptosis, Rho GTPase cycle, fatty acid metabolism, etc. The candidate genes of UI were involved in antigen processing, cellular senescence, macroautophagy, gluconeogenesis, etc. Thus, RM and UI may be concerned with abnormalities in immune response, energy metabolism, cellular function, etc. The underlying mechanisms of these two clinical phenotypes may have some similarities, but there are still many differences (Fig 2. A, B).

In addition, significant dysregulation of immune-inflammatory factors was found in the endometrium of RM and UI (Supplementary Fig. 1A, B). Furthermore, GSEA revealed the increased and decreased activity of inflammatory response in RM and UI, respectively (Supplementary Fig. 1C, D).

### 3.3 The alteration of immune cell abundance in RM and UI

**Figure 3:**
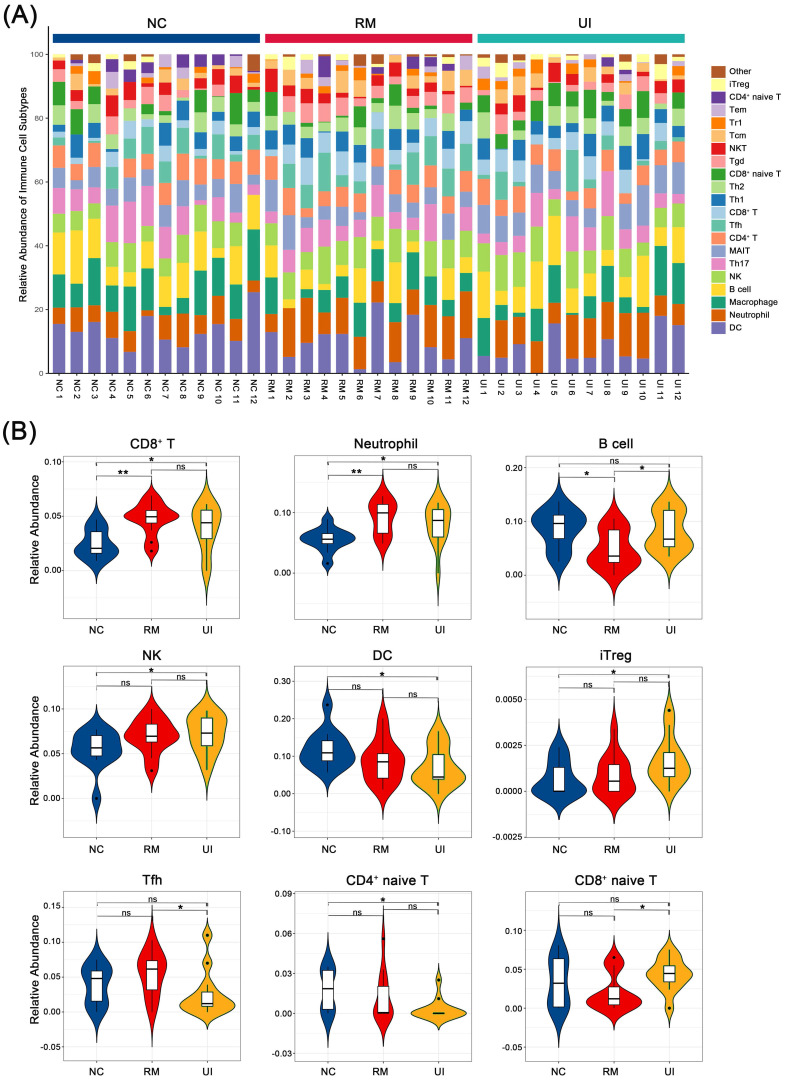
**Composition of infiltrated immune cells between case group and control.** (A) Stacked bar chart showing the composition of infiltrated immune cells. (B) Violin diagram showing significant difference in immune cell types. (NC: n = 12; RM: n = 12; UI: n = 12) (* indicates P < 0.05; ** indicates P < 0.01, ns indicates no significant difference)

We evaluated the relative abundance of 23 immune cells in the endometrium of normal, RM, and UI women, of which nine were identified as being different between the three groups: CD8+ T, neutrophil, B cell, NK, DC, iTreg, Tfh, CD4+ naïve T, and CD8+ naïve T (Fig. 3A). Among them, CD8+ T and neutrophil were significantly higher in both the RM (P < 0.05 for each of them) and UI (P < 0.01 for each of them) groups than in the normal group. The level of B cell was lower in the RM group (P < 0.05), while the level in the UI group was similar to that in the normal group. NK cell was significantly higher in the UI group compared to the normal group (P < 0.05), and we noted that there seems to be a similar trend of increased NK cells in the RM group, albeit not significant (P = 0.07). The relative abundance of DC cells was significantly lower than normal in the UI group (P < 0.05), while not significantly different from the RM group. In addition, we found that, unlike RM, the iTreg in the UI group was significantly higher than that in the normal group (P < 0.05) (Fig. 3B).

### 3.4 Screening for core genes of RM and UI

**Figure 4:**
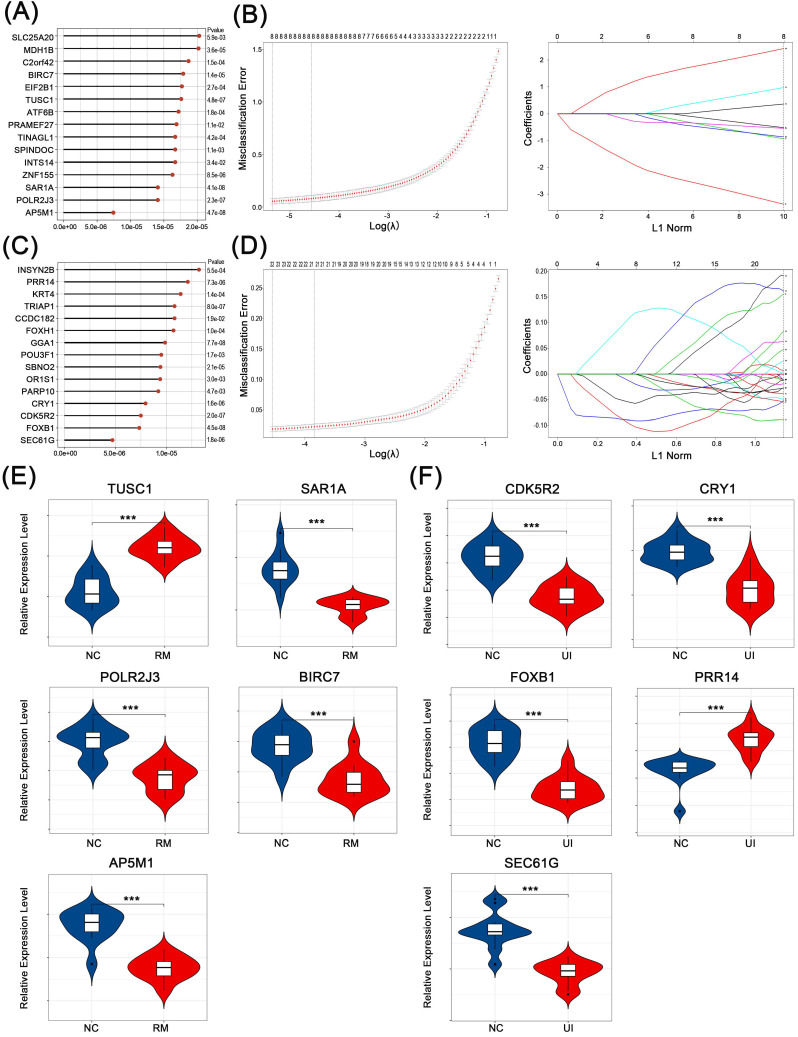
** Identification of core characteristic genes.** Top-ranked genes by their discriminant ability in the SVM algorithm step for RM (A) and UI (C). Coefficient profile plot showing the selection of the optimal parameter (lambda) in the LASSO model for RM (B) and UI (D). Identified core genes after integrating algorithm for RM (E) and UI (F). (NC: n = 12; RM: n = 12; UI: n = 12) (*** indicates P < 0.001)

The core genes that play a key role in the above-mentioned candidate genes for RM and UI (i.e., genes that effectively identify RM and UI) were selected in two steps. First, the classification analysis using SVM was performed and a total of 261 genes that contribute to the differentiation between normal and RM were identified (Fig. 4A). Similarly, 216 genes closely associated with RM were obtained (Fig. 4C). Subsequently, by lasso regression analysis, we targeted 5 core genes in each of the RM and UI groups (Fig. 4B, D). SAR1A, POLR2J3, BIRC7, and AP5M1 were lower in the RM group compared to the normal group, while TUSC1 was higher (P < 0.001 for each of them) (Fig. 4E). The gene with increased expression in the UI group was PRR14, and those with decreased expression were CDK5R2, CRY1, FOXB1, and SEC61G (P < 0.001 for each of them) (Fig. 4F). Apparently, there is no intersection between the key genes of RM and UI. Integrating these two approaches, we identified the importance of these core genes among all candidate genes, and therefore they were most likely being key links in the pathogenesis of RM and UI.

### 3.5 Diagnostic value of core genes in the endometrium of RM and UI

**Figure 5:**
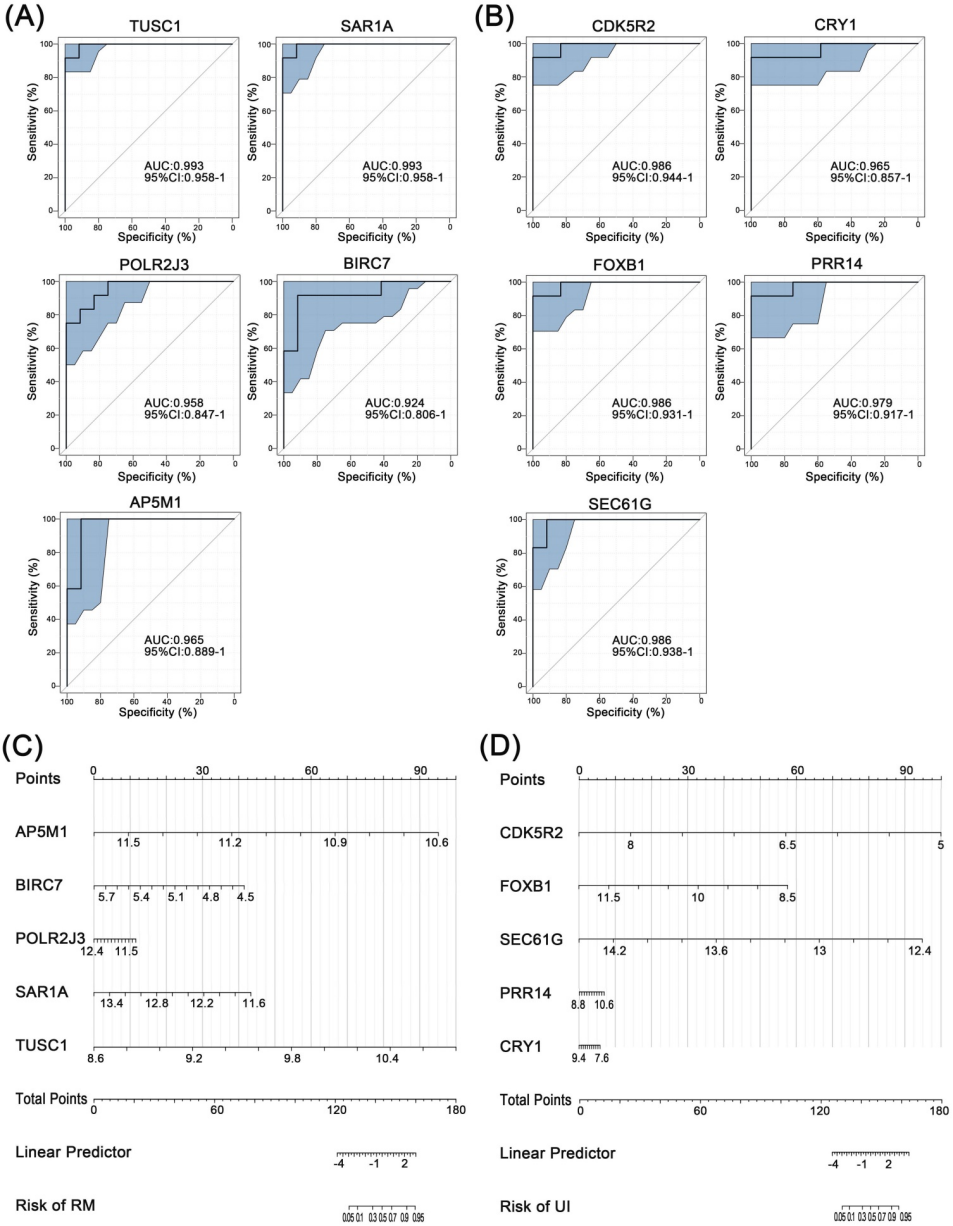
** Separating capability of the core characteristic gene model.** ROC curve of the diagnostic signature in the training group for RM (A) and UI (B). Nanogram of the core characteristic gene model for RM (C) and UI (D). (NC: n = 12; RM: n = 12; UI: n = 12)

Further, ROC curve analysis was used to assess the discriminatory ability of these core genes for clinical phenotypes. For RM, the AUC values of all five core genes were greater than 0.9, with the highest being TUSC1 (AUC=0.993) (Fig. 5A). Similarly, the AUC of all five core genes of UI reached above 0.9 (Fig. 5B). Finally, the nomogram models were constructed through multifactorial logistic regression analysis, to quantitatively estimate the effect of core genes on the onset of RM and UI (Fig. 5C, D).

### 3.6 Validation of candidate genes in RM and UI

We performed internal and external validation to confirm the reliability of these genes as core genes for RM and UI, respectively.

**Figure 6:**
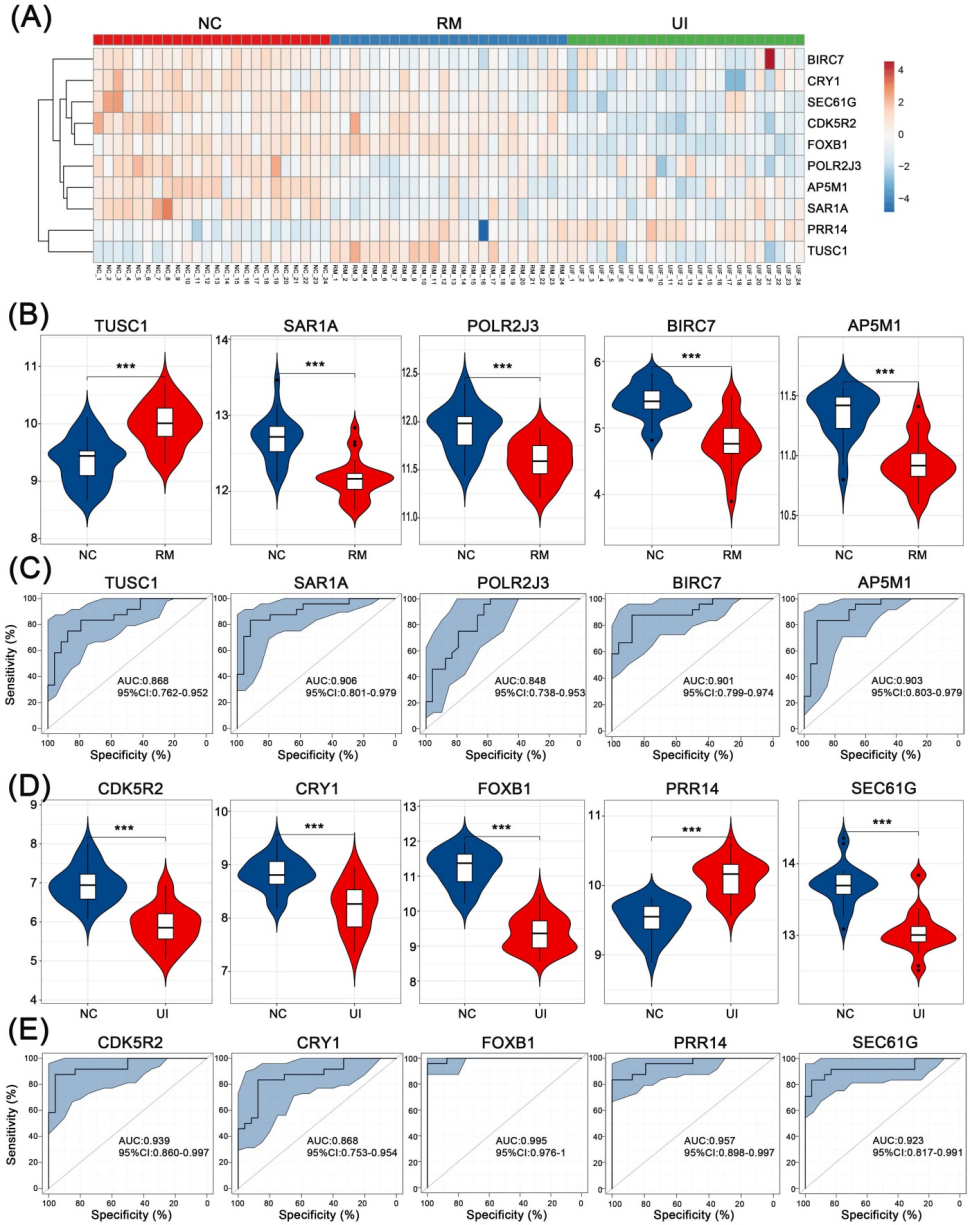
** Internal validation of the candidate genes in RM and UI.** (A) Heatmap of the core genes expression in each group. Expression of RM core genes (B) and UI core genes (D) in their respective group. ROC curve of core genes of RM (C) and UI (E). (NC: n = 24; RM: n = 24; UI: n = 24) (*** indicates P < 0.001)

For internal validation, data of 24 NC, 24 RM, and 24 UI samples were analyzed, and the overall expression of these core genes is shown in Fig. 6A. Consistent with the above results, all four were lower except for TUSC1 which was higher in the RM group (P < 0.001 for each of them) (Fig. 6B). Except PRR14 was higher in the UI group, the other four were lower (P < 0.001 for each of them) (Fig. 6D). Furthermore, ROC analysis showed that the diagnostic value of these core genes for RM and UI was high even in a larger sample size. For RM, the AUC ranged from 0.848 to 0.906, while for UI, the AUC ranged from 0.868 to 0.995 (Fig. 6C, E).

**Figure 7:**
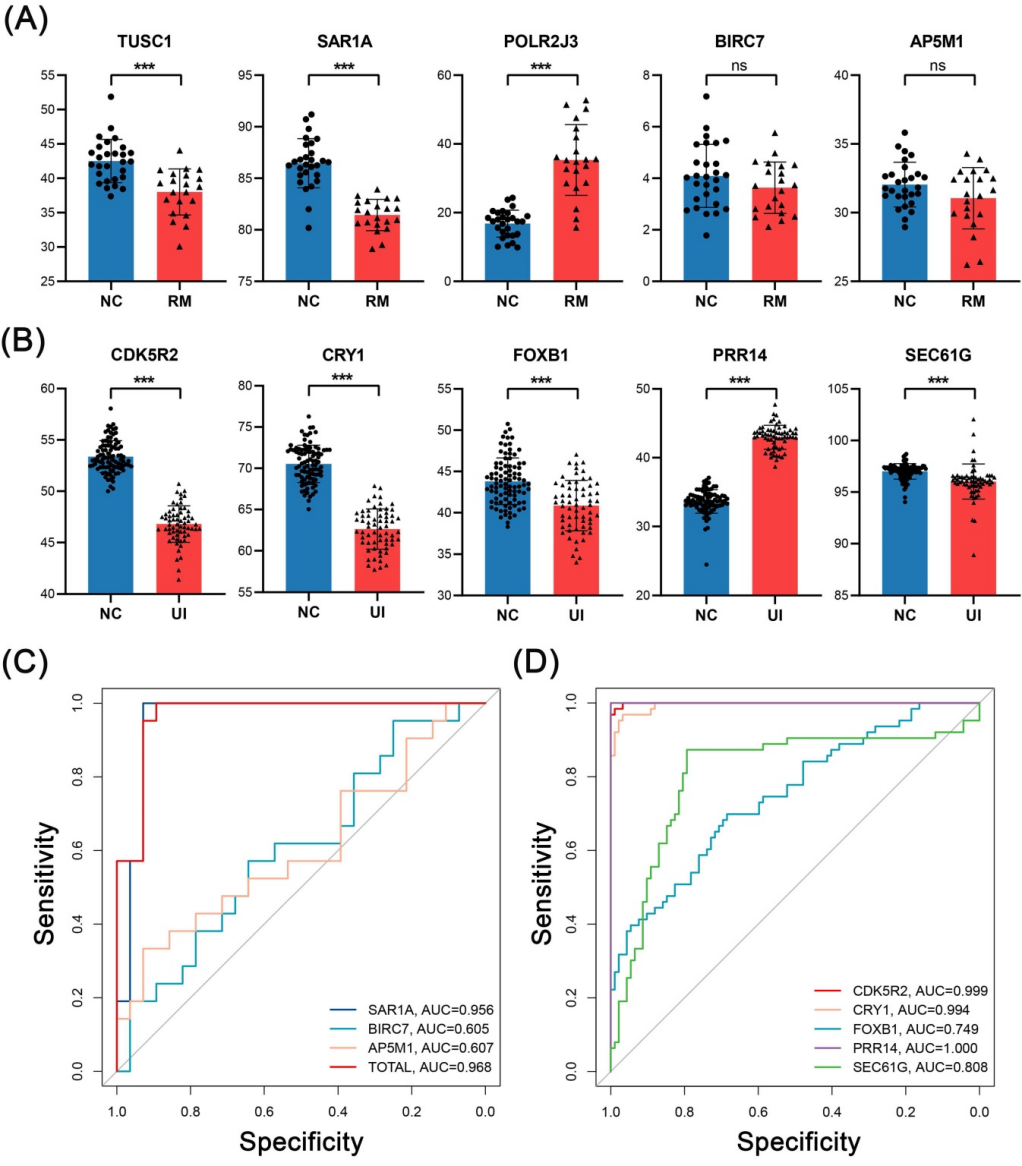
** External validation of the candidate genes in RM and UI.** Expression level (A) and ROC curve (C) of RM core genes (NC: n = 28; RM: n = 21). Expression level (B) and ROC curve (D) of UI core genes (NC: n = 92; RM: n = 63). (*** indicates P < 0.001, ns indicates no significant difference)

In external validation, the levels of RM core genes were analyzed in data from 28 NC and 21 RM samples, and levels of UI core genes were analyzed in data from 92 NC and 63 UI samples. SAR1A (P < 0.001), BIRC7 (P =0.16), and AP5M1 (P = 0.07) were decreased in the RM group, which is the same as the findings of internal validation. However, TUSC1 (P < 0.001) was decreased while POLR2J3 (P < 0.001) was increased in the RM group (Fig. 7A). So, we did not perform ROC analysis for these two genes, and even so, the combined AUC of SAR1A, BIRC7, and AP5M1 for RM identification reached 0.968 (Fig. 7C). In addition, the alterations of UI core genes (P < 0.001) were in line with that observed in internal validation and with even higher AUC values (Fig. 7B, D).

Thus, these results confirm the ability of these core genes as key discriminatory molecules for RM and UI, respectively.

## 4. Discussion

### 4.1 Progress and result of our study

In this study, we extracted transcriptome sequencing data from endometrial specimens from patients with RM and UI and normal control women at the mid-secretory phase of the menstrual cycle, a short suitable period of implantation (GSE165004). First, we used multiple biological information analysis methods to screen out the gene populations most related to the phenotypes of these two diseases, and from this, we mined that there is a wide correlation between them in the molecular pathways represented by RNA metabolism, signaling by TGFB family, etc. Subsequently, based on these molecular signatures and the immunopathological background of UI and RM, we assessed the abundance of major immune cells in the endometrium and found that CD8+T cells and neutrophils were found more abundant in RM and UI endometrium. Finally, the core genes, which had a good ability to identify the two diseases were screened out using comprehensive statistical analysis, and reliability was validated by additional data. Our study screened possible endometrium pathogenic genes in early pregnancy failure and discussed their possible effects on the disturbance of endometrial immune homeostasis, paving the way for further research on the mechanism.

### 4.2 Key results of our study, and the association with the current theory

From the multiple common function terms of our gene function annotation enrichment data, there are many similarities in the pathogenesis of both diseases (Fig. 2). In our data, RNA metabolism and membrane trafficking were the main common enrichment terms of both diseases. In contrast to the indirect regulation of gene expression by the transcription factor, the metabolic process of RNAs including synthesis, folding, modification, and degradation affect gene expression and function state more directly, which provides a new way of interpreting a variety of complex diseases 40, 41. Correspondingly, evidence from in vitro studies indicates that RNA metabolism is also involved in the occurrence of miscarriage, for example, miR-27a-3p induced degradation of USP25 inhibits the invasive ability of trophoblast cells 42. Non-coding RNA mediated RNA degradation also regulates human decidualization progress and affects uterine receptivity, which may be one of the mechanisms of infertility 43. Our results indicated that a large number of RNA metabolism genes were abnormally expressed in the endometrium of RM and UI, highlighting the key role of RNA-associated epigenetic mechanisms in endometrial function. However, the specific process of this regulation needs to be demonstrated in more detailed experiments. In addition, abnormality in membrane trafficking function seems to be another key common change occurring in the endometrium of RM and UI. This change in membrane trafficking function may affect endometrial function in two ways. On the one hand, as previously mentioned, endometrium stromal cells transform into secretory decidua cells during decidualization, providing essential nutrients for embryos that have not yet established blood supply 9. Therefore, the abnormal membrane transport function makes the embryo unable to obtain the necessary nutrient supply and to adapt to the early rapid division and differentiation, resulting in the delay of embryonic development and even death. On the other hand, abnormal membrane trafficking may also affect cell signal transmission in the maternal-fetal interface, especially the immunosuppressive signal, which is very important for the smooth progress of a pregnancy. A recent study found that placenta-derived extracellular vesicles contain a large number of soluble TNF-α receptors that promote the secretion of cytokines, especially interleukin-8 by decidual stromal cells, and may be a component of a successful pregnancy 44. Such evidence on cellular signaling of the placenta and decidua at the maternal-fetal interface was well-reviewed 45. Interestingly, immune-related pathways, such as SARS-CoV infections, ISG15 antiviral mechanism, and neutrophil degranulation, were also enriched in our results, which indicated a disruption of immune status in endometrium and inspired our interest in the immune infiltration in tissues. Therefore, we then calculated the relative ratio of immune cells in the tissue.

Consist with the reported elevated level of CD8+T cells in scRNA-seq data of RM decidua 24, our results showed that endometrium of both RM and UI contained more CD8+T cells and neutrophils, two important immune cells that can recognize exogenous signals (Fig. 3). The main function of CD8+T cells is to recognize non-autogenic antigens and kill non-autogenic and infected cells, however, the role of CD8+T cells in the early trimester remains largely unknown. A recent study indicated that depletion of CD8+T cells, especially fetal antigen-specific CD8+T cells, is beneficial to the maintenance of maternal-fetal pregnancy 46. The elevated level of effector memory CD8+T cells that do not express the inhibitory receptor PD1 was related to the occurrence of miscarriage and preeclampsia with a potential mechanism of immune activation and rejection of fetal antigen 47. Meanwhile, another study also indicated that the reduction of decidual CD8+T cells expressing the co-inhibitory molecules TIM3 and CTLA4 was associated with miscarriage 48. Thus, in summary, the appropriate number of CD8+T cells in the endometrium and their inhibitory functional phenotype are extremely important for the maintenance of pregnancy. On the other hand, according to current knowledge, although neutrophils are not involved in physiological pregnancy, they are both markers of infection as CD8+T cells. However, it is puzzling that the patients from whom these samples were derived do not have any known signs of infection. Autoimmunity may explain this non-infectious inflammation, as the induction of anti-phospholipid antibodies to complement C5a causes neutrophils to express tissue factors that aggravate decidual inflammation and lead to miscarriage 49. The formation of neutrophil extracellular traps (NETs) after apoptosis has also been suggested to be an important mechanism in many autoimmune diseases 50. Besides, the infiltration of neutrophils in the endometrium with an elevated level of cytokines and chemokines is also an important sign of the onset of menstruation suggesting a possible miss of implanting window, which is consistent with the dysregulated menstrual cycle in infertile women 51. In this regard, although the exact cause of neutrophilic infiltration in RM and UI is not clear, it appears to be more harmful than beneficial to implantation. A combination of autoimmune tests, estrogen-progesterone levels, and ovulation tests may shed some light on the cause of this inflammatory infiltration. Overall, our results suggest a potentially non-infectious inflammatory manifestation in the endometrium of RM and UI (Supplementary Fig. 1). Clearly, such infiltration of neutrophils and cytotoxic T cells is lethal for embryo implantation, however, the cause of this inflammatory manifestation is unknown.

Besides, elevated NK and iTreg cells were also observed in the endometrium of RM and UI in our data though there was no statistical significance in the RM group (Fig. 3B). NK cells are a dominant subset of immune cells in decidua during pregnancy with different phenotypes, and they are also the focus of studies on maternal-fetal immune tolerance. Research indicated that, on the one hand, NK cells promote implantation, placenta formation, and spiral artery remodeling, yet, on the other hand, overactivated NK cells can enhance the toxic effect on the embryo and cause miscarriage 12 Meanwhile, Tregs are a subset of pregnancy protective cells, belonging to a subset of special immunosuppressive CD4+T helper cells, which exert the inhibitory function in early pregnancy and can directly inhibit the killing of embryos by effector T cells, and secrete a variety of immunosuppressive factors to regulate other immune cells 52. The increase of both NK and iTreg seems to be an adaptive compensation of the disturbed immune balance to maintain a receptive environment, but it is difficult to suppress the damage caused by immunotoxicity of CD8+T cells and neutrophils to the embryo, which caused the occurrence of RM and UI finally. Furthermore, from the overall point of view, the proportion of immune cells in the UI group was more erratic than in the RM group including DC cells in addition to the cell types mentioned above. All this may partly explain why pregnancy failure occurs earlier in UI than in RM, as this severe immune cell disorder completely disrupts the normal endometrial immune microenvironment, resulting in barriers to embryo implantation that cannot be sustained even to the onset of early pregnancy.

The results of our immune calculation section showed that the number of immune cells in the endometrium of RM and UI patients was disturbed, which was characterized by infiltration of CD8+T cells, neutrophils, NK cells, and Treg cells (Fig. 3). Nevertheless, in summary, maternal-fetal interface immune balance is a complicated problem, not only related to the number of immune cells, the function of the state, is also closely related to cell crosstalk, our results reflect the RM and UI in the endometrial disorder of immune environment, but the disorder of detailed characteristics and internal contact need more research to prove.

Given the immune phenotypic similarities between the two diseases, we wondered if this similarity was also expressed at the molecular level, so we used statistical algorithms to screen out the core characteristic genes of the two groups. In RM endometrium, TUSC1 was at a higher level while SAR1A, POLR2J3, BIRC7, and AP5M1 were at a lower level (Fig. 4E). The functions of these genes are consistent with our results of functional annotation enrichment. It has been reported that SAR1A, as a precondition, is necessary for the assembly of the coat protein complex II (COPII) in eukaryotes, which is an endoplasmic reticulum selectively exported protein and lipid transport structure 53. COPII is a protein complex that determines the location of endoplasmic reticulum vesicle germination, and this is an important step in the process of cell secretion 54. This is consistent with our previous speculation that the loss of secretion of glycogen and signaling proteins by decidual stromal cells, as well as the loss of secretion of immune cells, especially NK cells inhibitory cytokines, will have a significant impact on early pregnancy. AP5M1, originally named MUDENG, has been reported to induce toxic T cell death and is widely expressed in a variety of tissues. Therefore, the decrease of AP5M1 may account for the increased number of CD8+T cells in the endometrium. On the contrary, BIRC7, a member of the inhibitor of the apoptosis protein family, was reported to protect melanoma cells against NK cell-induced apoptosis 55. Currently, the interaction of BIRC7 and NK cells in endometrium/decidual remains unknown. Considering the decreased level of BIRC7 and slightly increased number of NK cells in the RM group which leads to the occurrence of miscarriage, we attribute that BIRC7 in decidua meconium may possess the ability to prevent NK cells from killing embryonic and extraembryonic cells. In addition, POLR2J3 plays a role in RNA synthesis as a component of RNA polymerase II, meaning that it is upstream of multiple molecular and biological processes 56. Significant dysregulation of POLR2J3 in RM was found in different datasets (P <0.001), although the trends were contrary (Fig. 4E, 6B, 7A). We speculate that up- or down-regulation of this gene may promote RM by affecting different downstream biological processes, which requires further investigation. The last core gene, TUSC1, has good clinical predictive value in gastric cancer and hepatocellular carcinoma, and has been reported to inhibit the proliferative ability of tumor cells 57-59. So, it may have the same inhibitory effect on endometrial cells. Interestingly, the TUSC1 gene polymorphism has been proved to be strongly associated with male infertility, but its role in female reproductive health is still unknown and worthy to research 60.

In UI endometrium, PRR14 was at a higher level while CDK5R2, CRY1, FOXB1, and SEC61G were at a lower level (Fig. 4F, 6D, 7B). It was reported that PRR14 is a novel agonist of the PI3K pathway and cells overexpressing PRR14 show enlarged size and activation of the PI3K pathway 61. Meanwhile, PI3K/Akt pathway is involved in many processes and signaling pathways in different tissues, and its deregulation is involved in many pathological occurrences. It is also an important regulatory pathway for decidualization progress, and the most significant morphological change of endometrial cells is also the increase in cellular size 62. Yet, the significance of PRR14 elevation in decidualization and UI endometrium remains to be proved experimentally. More interestingly, CRY1 has also been identified as a characteristic gene for UI and is downregulated in the endometrium. CRY1 is a light-independent inhibitor of the mammalian CLOCK-BMAL1 complex, which controls the expression of rhythm genes in human 63. The important role of circadian rhythm in female infertility has been emphasized in recent years 64, 65. Evidence indicated that improvements in circadian rhythm can restore reproductive potential in mice 66. Given the influence of circadian rhythm on reproductive function, the role of the CRY1 gene in the endometrium would be an interesting topic. Moreover, FOXB1 is a member of the FOX gene family, and the human FOX gene family is believed to play a decisive role in the differentiation of Treg cells, especially FOXO1. However, the function of FOXB1 has rarely been described, but whether FOXB1 is involved in the differentiation of T cells in the endometrium is still worth further study. The SEC61G gene is a component of the SEC61 channel-forming translocon complex, which mediates the transport of signal peptide precursors in the endoplasmic reticulum 67, which is consistent with the membrane transport in the result of functional enrichment, suggesting the possible abnormal transport of signal peptide in the endometrium.

Overall, although no same core molecule was identified in both diseases, the core genes of the two groups execute a similar function, especially the cellular secretion and regulation of immune cell survival, both of which were essential for endometrial immune state transformation, may be the mechanism of RM and UI. However, the role of these genes in endometrium and pregnancy failure has not yet been elucidated.

### 4.3 Our characteristic and insufficiency

Our work provides some insights for further understanding of female endometrium, especially the immune -status and molecular expression patterns in pathological endometrium. We hypothesize that endometrial dysfunction is a shared mechanism of early pregnancy failure in women. The endometrium of RM and UI is described from phenotypic characteristics to molecular characteristics, and the connections and differences between the two are discussed. However, our work is still descriptive and speculative and requires further experimental confirmation. Although we have identified and verified the core characteristic molecules of the endometrium of the two diseases and found that they have excellent differentiating abilities, this still needs to be validated in a larger population. In addition, as described above, scRNA-seq has revealed the cellular and molecular landscape of RM decidua in early pregnancy, and this technology is providing a deeper and more comprehensive understanding of various diseases than traditional whole tissue -omics. Thus, it's worthy performing scRNA-seq on the mid-secretory endometrium, a highly heterogeneous tissue, to gain insight into the pathological basis of RM and UI in the future.

## 5. Conclusion

In conclusion, we analyzed endometrial transcriptome data in women with RM and UI diseases, identified phenotypic genes by WGCNA, and annotated the function of these genes. The proportion of immune cells in each tissue was then assessed and the similarities and differences between the two were discussed. Finally, we identified the core characteristic genes of both diseases and evaluated their ability to recognize the disease. In essence, our work delineates the molecular signatures of RM and UI in the endometrium, and by annotating these signature molecules in combination with reported evidence, it is possible to speculate on pathological endometrium dysfunction, such as transportation disorders and immune disorders. This provides exploratory biological data to further understand the mechanisms of early pregnancy failure in human women.

## Supplementary Material

Supplementary figure.

## Abbreviations

RM: Recurrent miscarriage
UI: Unexplained infertility
NK: Natural killer
WGCNA: Weighted correlation network analysis
GEO: Gene Expression Omnibus database
TOM: Topological overlap matrix
SVM: Support vector machines
NETs: Neutrophil extracellular traps
COPII: Coat protein complex II
