# Supplementary material for: Investigation and Validation of Molecular Characteristics of Endometrium in Recurrent Miscarriage and Unexplained Infertility from a Transcriptomic Perspective
Source: Int J Med Sci. 2022 Mar 6;19(3):546–62. doi: 10.7150/ijms.69648 (PMC8964333; doi:10.7150/ijms.69648)
Supplement: Supplementary file 1 — Supplementary figure. [file ijmsv19p0546s1.pdf]

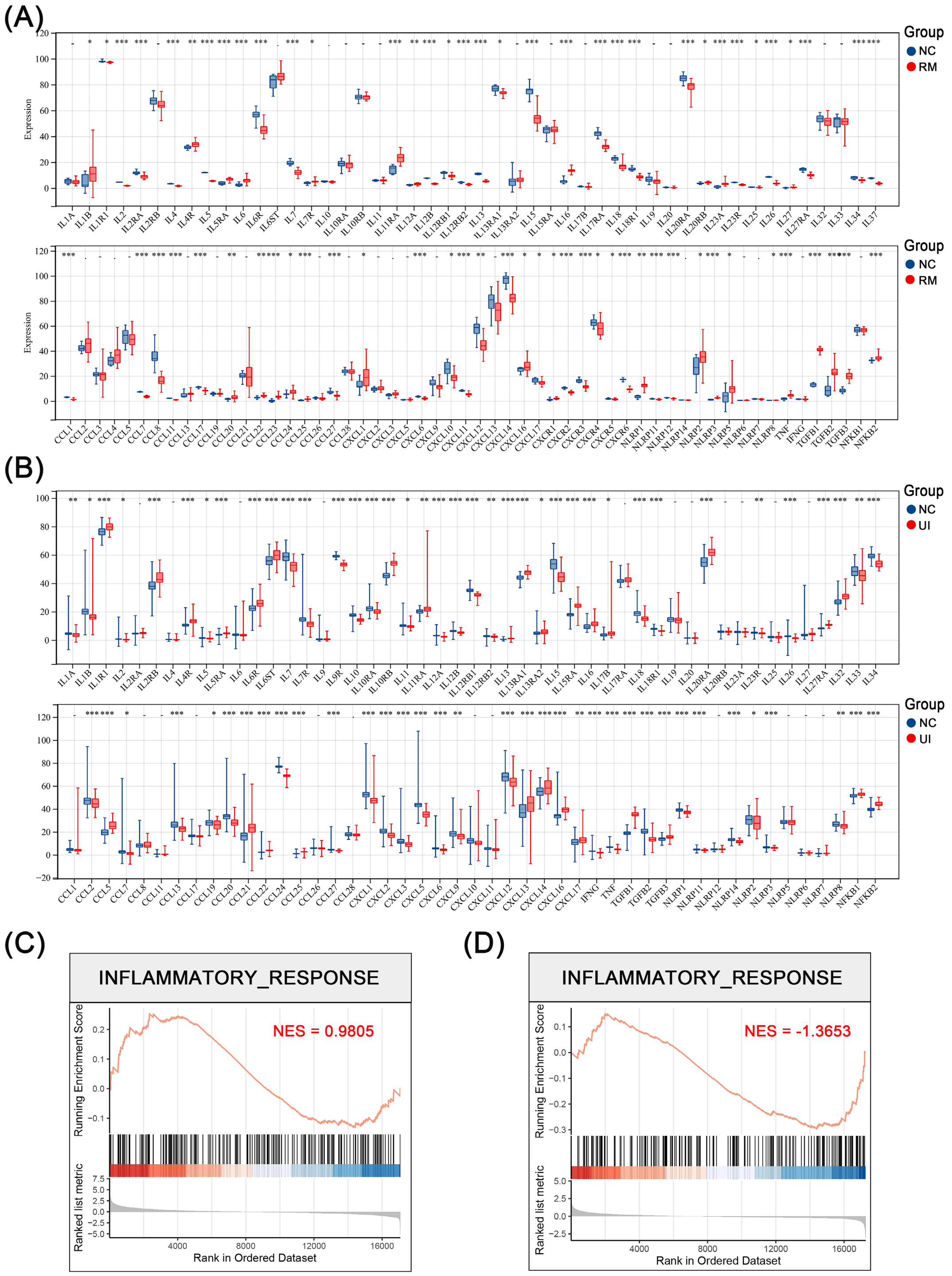

Supplementary Figure 1: The immune-inflammatory disorders in RM and UI.

The changes of the expression level of immune-inflammatory factors in RM (A) (NC: n = 8; RM: n = 21) and UI (B) (NC: n = 92; RM: n = 63). The activity of inflammatory response was evaluated by GSEA in RM (C) and UI (D). (* indicates P

< 0.05; ** indicates $P < 0.01$, *** indicates $P < 0.001$, ns indicates no significant difference)
